# Acute myocardial infarction with “wrap around” right coronary artery mimicking Takotsubo cardiomyopathy: a case report

**DOI:** 10.1186/s12872-016-0249-8

**Published:** 2016-04-22

**Authors:** Hiroki Shibutani, Yuzo Akita, Kotaro Yutaka, Satoshi Yamamoto, Yumie Matsui, Masahiro Yoshinaga, Masahiro Karakawa, Yasukiyo Mori

**Affiliations:** Division of Cardiology, Osaka Saiseikai Izuo Hospital, 3-4-5 Kitayama, Taisho-ku, Osaka 551-0032 Japan; Division of Nephrology, Osaka Saiseikai Izuo Hospital, 3-4-5 Kitayama, Taisho-ku, Osaka 551-0032 Japan

**Keywords:** Ischemic heart disease, Acute myocardial infarction, Takotsubo cardiomyopathy, Apical ballooning syndrome, Coronary artery anomaly

## Abstract

**Background:**

Takotsubo cardiomyopathy (TC) is a cardiomyopathy that shows distinctive clinical conditions first described more than 20 years ago. Because clinical features of TC mimic those of anterior acute myocardial infarction (AMI), the differential diagnosis is important in selecting the appropriate treatment strategy in the acute phase. But it was difficult to differentiate those two diseases because the TC-like findings; such as the electrocardiogram (ECG) changes and left ventricular wall motion abnormality can occur in AMI especially with the anatomical variance of the coronary artery.

**Case presentation:**

A 63-year-old man was admitted due to sudden onset of chest pain and was in a cardiogenic shock state. His ECG showed ST-segment elevation in precordial (V2–6) and inferior leads (II, III, and aVF) and ST-segment depression in lead aVR. Blood biochemistry showed that cardiac enzymes were not elevated. Ultrasonic cardiography showed that the left ventricular apical level was akinetic, papillary muscle level was severely hypokinetic, and basal level was hyperkinetic, mimicking TC. However, coronary angiogram showed total occlusion of his right coronary artery wrapping around the cardiac apex. Successful percutaneous coronary intervention reversed his critical status.

**Conclusion:**

To our knowledge, the present case is the first report described AMI with wrap-around RCA, mimicking TC. Although TC is increasingly recognized as a true but relatively infrequent clinical entity, it is still important to carefully rule out obstructive coronary artery disease.

## Background

Takotsubo cardiomyopathy (TC) is a cardiomyopathy that shows distinctive clinical conditions first described more than 20 years ago [1]. The ultrasonic cardiography (UCG) or left ventriculogram of TC shows transient left ventricular dysfunction apical ballooning (a round bottom and narrow neck), the shape of which looks a ‘Takotsubo’, a vessel that is used in Japan for trapping octopi. Because the clinical features of TC mimic those of anterior acute myocardial infarction (AMI), it’s sometimes difficult to distinguish TC from AMI. However, the differential diagnosis between two diseases is quite important in selecting the appropriate treatment strategy, especially in the acute phase. Some studies reported that the standard 12-leads electrocardiogram (ECG) findings on admission can help to differentiate these two diseases [2, 3]. For example, the absence of reciprocal changes, absence of abnormal Q waves, the distribution of ST-segment elevation and so on show high sensitivity and specificity for diagnosing TC. But it’s not practical, in other words, these ECG findings are not enough certainty to preclude the need for cardiac catheterization if not 100 % predictive accuracies. Because in AMI reperfusion therapy is required as soon as possible, we should not diagnose as TC without coronary angiogram (CAG).

In the present case, it was difficult to differentiate those two diseases. The initial findings on the ECG, UCG and the blood test were enough to suspect TC. However, as the patient’s status was relatively severe, we performed emergency CAG in order to evaluate his coronary artery and differentiate AMI.

## Case presentation

**Fig. 1:**
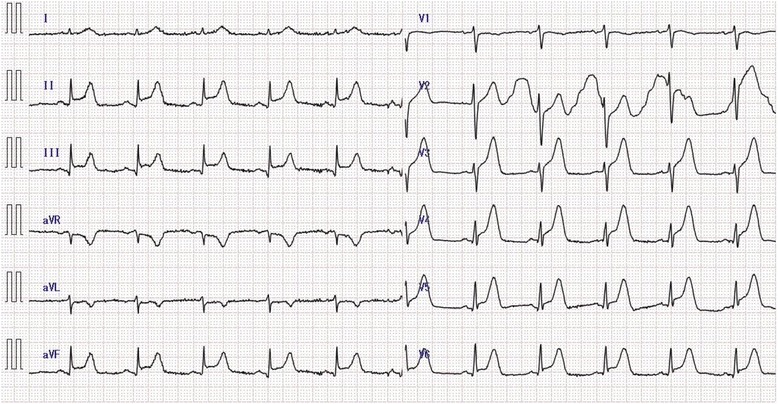
12 leads electrocardiogram (ECG) obtained on arrival. The ECG showed ST-segment elevation in the precordial (V2–6) and inferior leads (II, III, and aVF) and ST-segment depression in aVR

**Fig. 2:**
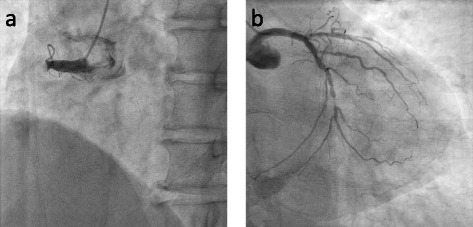
Emergency coronary angiogram of **a** the right coronary artery (RCA) in left anterior oblique (LAO) view and **b** the left coronary artery (LCA) in right anterior oblique (RAO) view. The total occlusion of the proximal RCA with a thrombus and the total occlusion of the proximal left anterior descending artery (LAD) were found

A 63-year-old man was transported to our hospital via an ambulance due to sudden onset of chest pain. He had untreated hypertension for the past 2 years. On arrival, he demonstrated pallor and incontinence of urine in the emergency department. He was in a cardiogenic shock state with a blood pressure of 90/40 mmHg and a heart rate of 60 beats/min. His ECG showed ST-segment elevation in the precordial (V2–6) and inferior leads (II, III, and aVF) and ST-segment depression in aVR (Fig. 1). We attempted emergent CAG to evaluate his coronary artery. During the preparations for catheterization we performed following tests. His blood levels of cardiac enzymes were not elevated, although the troponin T level was within the normal range, and the white blood cell (WBC) count was slightly higher than normal (Table 1). His UCG showed that the left ventricular apical level was akinetic, papillary muscle level was severely hypokinetic, and basal level was hyperkinetic, resembling TC. We treated with the intravenous administration of heparin (5000 units) as well as the oral administration of aspirin (200 mg) and clopidogel (300 mg) in the emergency room. And then he underwent an emergent CAG (Fig. 2), which showed the total occlusion of the proximal right coronary artery (RCA) with a thrombus, severe stenosis in the distal circumflex, and the total occlusion of the proximal left ascending artery (LAD). These findings led to a diagnosis of AMI.

**Table 1:** Laboratory data on admission

Parameter	Recorded value	(Standard value)
White blood cell count	9.0 × 10^3^/μL	(3.9–9.8 × 10^3^/μL)
Red blood cell count	5.06 × 10^6^/μL	(4.27–5.70 × 10^6^/μL)
Hemoglobin	15.2 g/dL	(13.5–17.6 g/dL)
Platelet count	225 × 10^3^/μL	(130–369 × 10^3^/μL)
Urea nitrogen	15 mg/dL	(8–15 mg/dL)
Creatinine	0.94 mg/dL	(0.61–1.04 mg/dL)
Sodium	142 mEq/L	(135–147 mEq/L)
Potassium	4.6 mEq/L	(3.6–5 mEq/L)
Creatine kinase	101 IU/L	(50–250 IU/L)
Creatine kinase MB	23 IU/L	(3–25 IU/L)
Aspartate aminotransferase	32 IU/L	(10–40 IU/L)
Alanine aminotransferase	19 IU/L	(5–45 IU/L)
Lactate dehydrogenase	346 IU/L	(115–245 IU/L)
Glucose	201 mg/dL	(70–109 mg/dL)
Hemoglobine A1C	5.2 %	(4.6–6.2 %)
C-reactive protein	0.07 mg/dL	(0–0.3 mg/dL)
Toroponin T	Negative	-

**Fig. 3:**
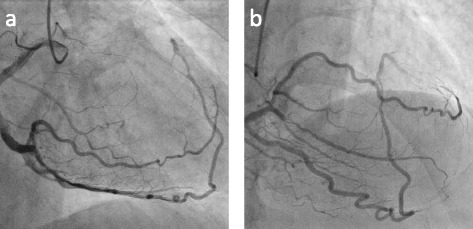
Coronary angiogram of the right coronary artery (RCA) after percutaneous coronary intervention (PCI). **a** Right anterior oblique (RAO) view, **b** RAO-cranial view. The right posterior lateral artery communicated with the diagonal branch and the right posterior descending artery communicated directly with the left anterior descending artery (LAD)

**Fig. 4:**
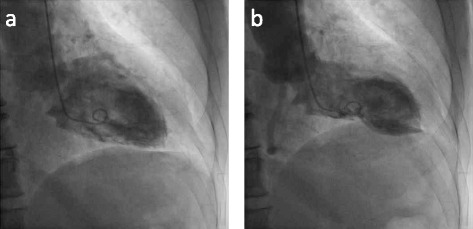
Left ventricular angiogram (RAO view) after PCI. **a** diastole, **b** systole. The wall motion abnormality of left ventricle mimicked Takotsubo cardiomyopathy

The culprit lesion was proximal RCA diagnosed from his CAG. We inserted an intra-aortic balloon pumping (IABP), and he was intubated using mechanical ventilation. Then, we performed percutaneous coronary intervention (PCI) of the proximal RCA. We aspirated the thrombus and implanted a coronary stent in the proximal RCA to provide TIMI-3 blood flow in the RCA. After stent implantation, his CAG showed that his right posterior lateral artery communicated with the diagonal branch and his right posterior descending artery communicated directly with the LAD (Fig. 3), and his left ventricular angiogram showed like TC (Fig. 4).

**Fig. 5:**
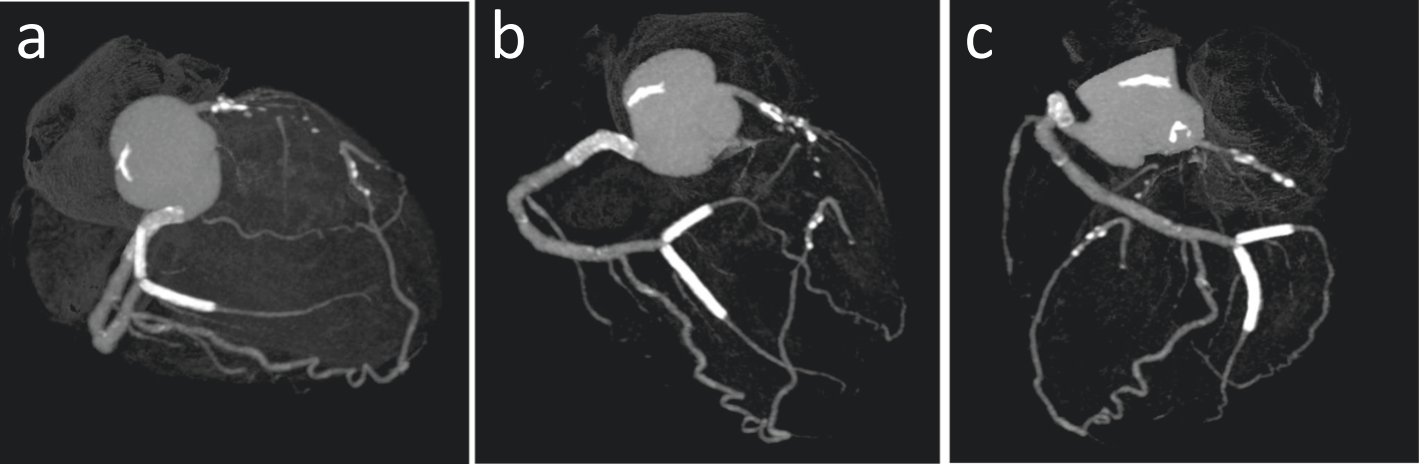
Coronary computed-tomography angiography (CTA). CTA was performed after elective percutaneous coronary intervention (PCI) to the distal branches of the right coronary artery (RCA) (#4:posterior lateral branch and atrio ventricular branch). **a** Right anterior oblique (RAO)-cranial view, **b** Anteroposteriol-cranial view, **c** Left anterior oblique (LAO)-cranial view. The right posterior descending artery communicated directly with the left anterior descending artery (LAD) through the apex

The patient was admitted to the intensive care unit and then proper ST-segment resolution was achieved. He was weaned from the IABP and was extubated for 3 days after the procedure. No major complications occurred. On day 23, he was discharged from our hospital after elective PCI to the distal branches of RCA posterior lateral branch and atrio-ventricular branch (Fig. 5).

## Discussion

The clinical features of TC mimic those of anterior AMI. Differential diagnosis is important in selecting the appropriate treatment, particularly in the acute phase. Although first described more than 20 years ago [1], understanding of the cause and mechanisms of TC is rudimentary. Among many theories proposed to explain its cause, the four most frequently studied aspects of TC are as follows: morphological characteristics of LAD [4], multiple coronary spasm, microcirculatory dysfunction, and catecholamine-mediated myocardial stunning [5].

We note two significant aspects of the present case. First, to avoid misdiagnosing AMI as TC, the anatomical variance of the coronary artery should always be considered [6]. TC was not accepted as a distinct clinical entity but rather a manifestation of a spontaneously aborted AMI [7]. A long LAD that extends past the apex and supplies the inferior wall of the left ventricle (LAD recurrent segment or wrap-around LAD) was observed in some TC cohorts [8, 9]. Although recent larger studies do not confirm that wrap-around LAD is the sole cause of TC, the end-systolic LV shape in AMI patients with complete occlusion of the proximal or middle portion of wrap-around LAD can be indistinguishable from that in TC [7]. Thus, the abnormal regional wall motion that extends beyond the distribution of a single coronary artery (apical ballooning) is not always diagnosed as TC [4].

The anatomical characteristics of the present case showed abnormal communication of the right posterior lateral artery with the diagonal branch as well as direct communication of the right posterior descending artery with the LAD. Total occlusion of the proximal RCA with total occlusion of his short LAD contributed to his acute coronary syndrome, and we diagnosed him with AMI. The frequency of such an abnormality appears to be low [10, 11]. Alternatively, the communications in the present case might be developed as the collateral circulation. Although we cannot rule out that possibility, the distal LAD filled via direct continuity from right posterior descending artery without any septal collaterals are also rare [12, 13]. Nevertheless, the blood flow from his RCA “wrapped around” the apex and supplied the inferior ventricle. The culprit lesion of this event was proximal RCA. Therefore, the left ventricular angiogram showed apical ballooning resembling TC.

Second, some studies reveal distinct differences between the ECGs of patients with TC or anterior AMI [2]. These differences will help differentiate TC from AMI. For example, TC is more frequently associated with the absence of reciprocal ST-segment depression in inferior leads and the absence of abnormal Q waves compared with anterior AMI [3, 14]. Moreover, these two diseases differ according to the frequencies of ST-segment elevation in all 12 leads. TC is associated more frequently with ST-segment elevation, particularly in –aVR (+30°) and less frequently with ST-segment elevation, particularly in V1.So the ST-segment shift in leads -aVR and V1 can help to differentiate TC from AMI. The lead -aVR faces the apical and inferolateral regions, and ST-segment elevation in -aVR in TC is thought to reflect the extensive distribution of wall-motion abnormalities centered around the apex. In the standard 12 leads, we can recognize as ST-segment depression in the opposing lead aVR (−150°). In contrast, the lead V1 faces the right ventricular anterior region as well as the septal region. The most likely reason for lower ST-segment elevation in V1 in TC is that wall-motion abnormalities in TC rarely extend to the region faced by V1 [15, 16]. In the report, the combination of the presence of ST-segment depression in aVR and the absence of ST-segment elevation in V1 identified TC with 91 % sensitivity, 96 % specificity, and 95 % predictive accuracy [16]. In anterior AMI, the perfusion range of the LAD usually does not extend to the regions around the apex; therefore, the prevalence of ST-segment elevation in –aVR is low.

The ECG findings of our present patient showed ST-segment elevation in precordial leads (V2–6) and inferior leads (II, III, and aVF), presence of ST-segment depression in aVR (−150°), and absence of ST-segment elevation in V1. These findings strongly suggested TC, but the final diagnosis was AMI because of his wrap-around RCA-perfused LAD regions as well as the TC-like abnormality with typical ECG changes of left ventricular wall motion. Therefore, these findings indicate the importance of administering a CAG.

## Conclusion

To our knowledge, the present case is the first report described AMI with wrap-around RCA, mimicking TC. Although TC is increasingly recognized as a true but relatively frequent clinical entity, it is still important to carefully rule out obstructive coronary artery disease. We should not diagnose as TC without CAG easily because the TC-like findings; such as the ECG changes and left ventricular wall motion abnormality can occur in AMI.

### Consent

Written informed consent was obtained from the patient for publication of this case report and any accompanying images. A copy of the written consent is available for review by the Editor of this journal.

## References

[1] Dote K, Sato H, Tateishi H, Uchida T, Ishihara M (1991). Myocardial stunning due to simultaneous multivessel coronary spasms: A review of 5 cases. J Cardiol.

[2] Kosuge M, Kimura K (2014). Electrocardiographic findings of takotsubo cardiomyopathy as compared with those of anterior acute myocardial infarction. J Electrocardiol.

[3] Ogura R, Hiasa Y, Takahashi T (2003). Specific findings of the standard 12-lead ecg in patients with 'takotsubo' cardiomyopathy: Comparison with the findings of acute anterior myocardial infarction. Circ J.

[4] Stiermaier T, Desch S, Blazek S, Schuler G, Thiele H, Eitel I (2014). Frequency and significance of myocardial bridging and recurrent segment of the left anterior descending coronary artery in patients with takotsubo cardiomyopathy. The Am J Cardiol.

[5] Pelliccia F, Greco C, Vitale C, Rosano G, Gaudio C, Kaski JC (2014). Takotsubo syndrome (stress cardiomyopathy): An intriguing clinical condition in search of its identity. Am J Med.

[6] Perlmutt LM, Jay ME, Levin DC (1983). Variations in the blood supply of the left ventricular apex. Invest Radiol.

[7] Ibanez B, Benezet-Mazuecos J, Navarro F, Farre J (2006). Takotsubo syndrome: A bayesian approach to interpreting its pathogenesis. Mayo Clin Proc.

[8] Ibanez B, Navarro F, Farre J (2004). tako-tsubo syndrome associated with a long course of the left anterior descending coronary artery along the apical diaphragmatic surface of the left ventricle. Rev Esp Cardiol.

[9] Migliore F, Maffei E, Perazzolo Marra M (2013). LAD coronary artery myocardial bridging and apical ballooning syndrome. JACC Cardiovasc Imaging.

[10] Yamanaka O, Hobbs RE (1990). Coronary artery anomalies in 126,595 patients undergoing coronary arteriography. Cathet Cardiovasc Diagn.

[11] Tuncer C, Batyraliev T, Yilmaz R (2006). Origin and distribution anomalies of the left anterior descending artery in 70,850 adult patients: multicenter data collection. Catheter Cardiovasc Interv..

[12] Levin DC (1974). Pathways and functional significance of the coronary collateral circulation. Circulation.

[13] Abu-Ful A, Margulis G, Ilia R (1995). Unusual coronary collateral circulation: filling of a totally occluded left anterior descending artery by direct continuity from a left posterior descending artery. A case report. Angiology.

[14] Raitt MH, Maynard C, Wagner GS, Cerqueira MD, Selvester RH, Weaver WD (1995). Appearance of abnormal q waves early in the course of acute myocardial infarction: Implications for efficacy of thrombolytic therapy. J Am Coll Cardiol.

[15] Wagner GS, Macfarlane P, Wellens H (2009). AHA/ACCF/HRS recommendations for the standardization and interpretation of the electrocardiogram: Part VI: Acute ischemia/infarction: A scientific statement from the american heart association electrocardiography and arrhythmias committee, council on clinical cardiology; the american college of cardiology foundation; and the heart rhythm society: Endorsed by the international society for computerized electrocardiology. Circulation.

[16] Kosuge M, Ebina T, Hibi K (2010). Simple and accurate electrocardiographic criteria to differentiate takotsubo cardiomyopathy from anterior acute myocardial infarction. J Am Coll Cardiol.

